# Path Renewal Method in Filtering Based Wireless Sensor Networks

**DOI:** 10.3390/s110201396

**Published:** 2011-01-26

**Authors:** Jin Myoung Kim, Young Shin Han, Hae Young Lee, Tae Ho Cho

**Affiliations:** 1 School of Information and Communication Engineering, Sungkyunkwan University, Suwon 440-774, Korea; E-Mails: kjm77@ece.skku.ac.kr (J.M.K.); yshan95@ewhain.net (Y.S.H.); 2 Embedded Software Division, Electronics Telecommunications Research Institute, Daejeon 305-700, Korea; E-Mail: haelee@etri.re.kr (H.Y.L.)

**Keywords:** filtering scheme, load balancing, sensor networks, path finding

## Abstract

In applications of wireless sensor networks, there are many security issues. Attackers can create false reports and transmit the reports to the networks. These false reports can lead not only false alarms, but also the depletion of limited energy resources. In order to filter out such false reports during the forwarding process, Ye *et al.* proposed the statistical en-route filtering (SEF). Several research efforts to enhance the efficiency of SEF have been made. Especially, the path selection method proposed by Sun *et al.* can improve the detection power of SEF by considering the information on the filtering keys of and distances of upstream paths. However, such selection mechanism could lead to favored paths in heavy traffic, which would result in unbalanced energy consumption. In this paper, we propose a path renewal method to provide load balancing for sensor networks in terms of energy consumption. In our method, a node renews its upstream path to save energy resources if the remaining energy of and the communication traffic of the node exceed some threshold values. We show the effectiveness of the proposed method in terms of balanced energy consumption and filtering power by providing simulation results.

## 1. Introduction

Recent advances in wireless communications and electronics have enabled the development of low-cost, low-power and multi-functional sensors that are small in size and communicate over short distances [1]. A wireless sensor network (WSN) is composed of a large number of small sensors with constrained energy, limited computation, communication range, and unchangeable battery power. Sensor nodes can be distributed in an outdoor environment to collect sensing data and forward it to base station via wireless channel [2–4]. Applications of WSNs range from indoor applications such as smart homes and health monitoring in a hospital to outdoor applications such as highway traffic monitoring, combat field surveillance, security and disaster management [5–8].

In many applications, WSNs are deployed in outdoor environments. Consequently, they are vulnerable to false data injection attacks [9] in which an adversary inject false sensing reports into the network, through compromised nodes, with the goal of deceiving the base station or draining the constrained energy of the nodes [10]. The statistical en-route filtering scheme (SEF) [9] can filter out forged reports during the forwarding process. In the scheme, for an event, sensing nodes collaboratively generate a report which contains message authentication codes (MACs) so that each MAC is generated from a node using its symmetric keys and represents its agreement on the report [11]. As a report is forwarded towards the base station over multiple hops, each forwarding node verifies the MACs carried in the report, checking if it has any of the keys used to generate those MACs. If it does not have any of those keys, the report is forwarded without verification. Therefore, the detection power of the SEF is affected considerably by the choice of routing path [12].

The path selection method (PSM) [12] was proposed to improve the detection power of SEF. In PSM sensor nodes evaluate the detection power of each incoming path from the base station and elect the most secure path for data transmission against false data injection attacks. In order to evaluate the path, each sensor node inserts additional information about filtering keys into a control message. However, such path selection based on the security power would make the most secure paths undergo heavy traffic so that the nodes along the paths would consume more energy resources. That is, the limited energy resources of the network would be spent in an unbalanced fashion, which could cause the decrease of the overall network lifetime.

In this paper, we propose a path renewal method (PRM) to prolong a network lifetime. While the energy consumption of each sensor node is basically proportional to data transmissions, events do not uniformly occur on a sensor field. Thus, we cannot predict the energy consumption patterns in the network. In the paper, we represent a WSN as a digraph (directed graph), and define a communication traffic model. Based on the model, we propose a fitness function for the renewal of routing paths. To show the effectiveness, we have compared the proposed method with the two existing methods, SEF and PSM, in terms of balanced energy consumption and reliability of data transmission by providing simulation results.

The remainder of the paper is organized as follows: Section 2 briefly explains the related works and the motivations of this work. Sections 3, 4, and 5 present a network model, the proposed path renewal method, and an evaluation function, respectively. Section 6 gives simulation results. Finally, conclusions and future works are covered in Section 7.

## 2. Related Works and Motivations

In this section, we review the two existing methods—SEF and PSM—and then explain the motivations of this paper.

### 2.1. Statistical En-routed Filtering Scheme (SEF)

SEF was the first scheme to address false data injection attacks in the presence of compromised nodes and it focuses on the detection of false event reports, which are known as false positive attacks, injected by compromised nodes. In SEF, the base station maintains a global key pool, which is divided into multiple partitions and every node loads a small number of keys from a randomly selected partition in the global key pool before it is deployed.

When real events occur, one of the detecting nodes is elected as the center-of-stimulus (CoS) node to generate a sensing report. The surrounding nodes, which detect the same event, produce MACs for the event, using their stored keys, and send them to the CoS which generates a sensing report using the collected MACs. This set of multiple MACs acts as the proof that a report is legitimate [9] after which points the CoS forwards the report toward the base station (BS) over multi hops. Each forwarding node verifies the correctness of the MACs carried in the report by using its keys. When the BS receives a report, it can verify all the MACs carried in the report because it has complete knowledge of the global key pool [9].

### 2.2. Path Selection Method (PSM)

In SEF, the detection power of false reports is affected considerably by the choice of the routing paths. In the worst case, forwarding nodes may not have any of the keys used in report generation so these forwarding nodes cannot verify any false reports.

In [12], authors proposed a path selection method (PSM) in order to improve the filtering power for false positive attacks. In PSM, routing paths are established by flooding with a control message [13,14] and can be selected with the consideration of the security level and the transmission distance. The control message contains information about the partition IDs of visited nodes and hop count. This information is used to evaluate the quality of the path.

### 2.3. Motivations

In PSM, after routing paths are established in the initial phase, each sensor node only sends data to designated sensor node (e.g., the most downstream nodes along the chosen path). Let a transmitting node be a sub-node and a receiving node be a super-node. In a PSM-based network, a single sub-node can be assigned to only one super-node or a single super-node can have multiple one sensor nodes (if it is on a ‘promising’ path). Thus, the super-node that has many sub-nodes will consume more energy than other super-nodes that have small number of sub-nodes. Therefore, the network lifetime will decrease due to such unbalanced energy consumption.

In this paper, we propose a path renewal method (PRM). After the routing paths are established, each super-node checks its remaining energy. If the remaining energy of its super-node is less than a pre-defined threshold value, one of super-node’s children (*i.e.*, sub-nodes) changes the routing path using PRM. That is, the sub-node chooses a new super-node. The super-node manages the list of its sub-nodes. The super-node sends an eviction message to the sup-node. The super-node selects the sub-node by considering the sub-node’s communication traffic. The detailed description is presented in section 4 and our network model is described in the next section.

## 3. Network Model

A wireless sensor network is composed of a base station and large number of sensor nodes. The network can be represented as a digraph (or directed graph) *G*. The graph *G* is defined as follows:

(1) $$\begin{array}{l} G=\left(V,E\right)\\ \text{where},\\ V=\left\{v_{1},v_{2},\ldots ,v_{n}\right\}\\ E=\left\{e_{1},e_{2},\ldots ,e_{m}\right\}\\ E\subset V\times V \end{array}$$

**Figure 1.**
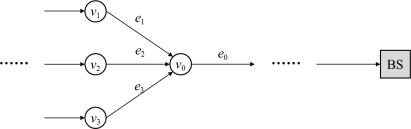
In-degree and out-degree.

In Equation (1), *V* is a set of vertices and each vertex denotes a sensor node. *E* is a set of edges and each edge denotes a link between vertices (*i.e.*, sensor nodes). For two arbitrary integers *i* and *j*, where *i* and *j* are less than *n*, *e_ij_* (∈*E*) indicates a communication link between vertex *v_i_* and *v_j_* (*v_i_*,*v_j_* ∈ *E*). An in-degree (and out-degree) is the number of inward (and outward) graph edges from a given graph vertex in the directed graph. Figure 1 shows the in-degree and out-degree.

In the figure, the in-degree and out-degree of *v*_0_ are 3 and 1, respectively. We denote that *v*_0_ is the super-node for nodes *v*_1_, *v*_2_ and *v*_3_. Also, nodes *v*_1_, *v*_2_ and *v*_3_ are sub-nodes of *v*_0_, respectively. Additionally, the number of the in-degree can be represented as an amount of communications.

In this paper, we propose a path renewal method to uniformly consume energy resources. In our proposal, each sensor node can know the amount of communications and remaining energy. In the figure, if the remaining energy of *v*_0_ is less than a threshold value, one of the sub-nodes searches a new super-node. Our proposal is briefly illustrated in the next section.

## 4. Path Renewal Method

In our network model, routing paths are established by the flooding of a control message. This fashion is commonly used in most routing protocols at the initial establishment of routing path. Similar to PSM, a control message includes information on the partition of the keys (PIK) and on hop counts from the base station (HC). Also, each node can know its own in-degree.

**Figure 2.**
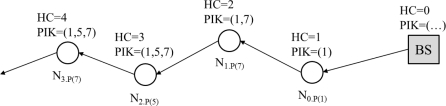
Flooding control message.

Figure 2 shows a propagation of a flooding message. In the figure, the node that received the message inserts its PIK to the message and forwards it to the next hop (toward terminals). Suppose *N*_2_ receives the control message including PIK of 1 and 7 from *N*_1_. When *N*_2_ sends the message to the next node, it inserts its PIK to the message. Here, given *N*_1_, *N*_1.PIK(5)_ implies that *N*_1_ stores PIK that is 5. *N*_3_ does not need to insert the PIK to the message since PIK in the message already has PIK(7).

After the paths are established, all nodes store their in-degree and the list of the sub-nodes by elapsed time. Each node manages the list. The list is comprised of IDs of sub-nodes and the number of data transmissions of each sub-node. For an arbitrary super-node *N*_sup_ and three sub-nodes *N*_sub.1_, *N*_sub.2_ and *N*_sub.3_, the process of path renewal is as follows:

In the table, TE, EM, and FM are threshold energy, eviction message, and fare message, respectively. Let *N*_sub.3_ have the highest number of the transmissions in the list. If the remaining energy of *N*_sup_ is less than TE, *N*_sup_ sends EM to *N*_sub.3_. EM includes the fitness value of *N*_sup_. *N*_sub.3_ finds a new super-node in the neighboring nodes. Each of the neighboring nodes sends its own fitness value to *N*_sub.3_. If *N*_sub.3_ finds a new super-node that has the highest fitness value, *N*_sub.3_ sends FM to *N*_sup_ and migrates to other super-node. After *N*_sup_ receives the RM from *N*_sub.3_, *N*_sup_ removes *N*_sub.3_ in the list.

## 5. Evaluation Function

To elect a new super-node, we define an evaluation function by considering HC, ID, EC and diversity of PIK. The evaluation function is defined as follows:

(2) F(n)=EC(n)+α⋅DPIK(n)

In Equation (2), the evaluation function consists of EC and DPIK. EC is energy consumption and DPIK is a diversity of PIK. Alpha is a security weight factor determined by the user. So, for an arbitrary sensor node n, EC and DPIK are defined as follows:

(3) $$\begin{array}{l} \mathrm{EC}\left(n\right)=\mathrm{HC}+\mathrm{ID}+\mathrm{RE}=\frac{1}{n_{\mathrm{HC}}\cdot E_{t}+\left(n_{\mathrm{HC}}-1\right)\cdot E_{r}+n_{\mathrm{ID}}\cdot \left(E_{t}+E_{r}\right)}+n_{\mathrm{RE}}\\ \mathrm{DPIK}\left(n\right)=\left|\mathrm{PKI}\left(n\right)\right|\\ \text{where},\\ E_{t}\text{ is energy consuption by a transmission}.\\ E_{r}\;\text{ is energy consumption by receiving data}.\\ \left|\mathrm{PKI}\left(n\right)\right|\text{ is a number of elements of }\mathrm{PKI}\left(n\right) \end{array}$$

In Equation (3), n_HC_, n_ID_ and n_RE_ are a hop count, in-degree and remaining energy of the node n respectively. It is clear that energy consumption is affected by hop count and in-degree. So, we can represent energy consumption of the node with consideration of n_HC_ and n_ID_. DPIK implies a diversity of partition information of key. In the equation, DPIK is a number of elements of PIK.

## 6. Simulation Results

A simulation was performed to compare the proposed PRM method with the existing SEF and PSM ones. A performance criterion is balanced energy consumption and success rate of data transmission. We also analyze the detection power of proposed method. In the simulation, the network consists of 1,000 nodes spread over a territory whose size is 100 ×120 m. The nodes are randomly deployed in the territory and the base station is placed at the end of the territory. Each sensor node takes 16.56 μJ/12.5 μJ to transmit/receive a byte, and each MAC generation consumes 15 μJ. The size of original report and of MAC is 24 and 1 bytes, respectively. There are 1,000 keys in the key pool, which is divided into 10 partitions.

**Figure 3.**
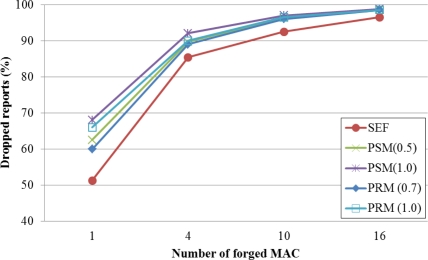
Ratio of filtered false reports with the security value (α).

Figure 3 shows a filtering rate for false reports with a security weight in case that the number of forged MACs per a report is 1, 4, 10 and 16. For the same network topology, routing paths on SEF, PSM and PRM are established, respectively. Then we generate false reports in the network. Assigned keys in each node are randomly generated with various seed values from 0 to 9. We calculate an average dropping rate for the false report.

In the figure, the proposed method is better than SEF but less efficient than PSM in terms of dropping ratio. The figure illustrates a similar performance for PRM and PSM. In PSM, each node chooses a super-node by considering information of keys on incoming path from the base station. In PRM, each node only has partition information of nodes within five hop counts, so the performance of the proposed method is a little less efficient than PSM.

**Figure 4.**
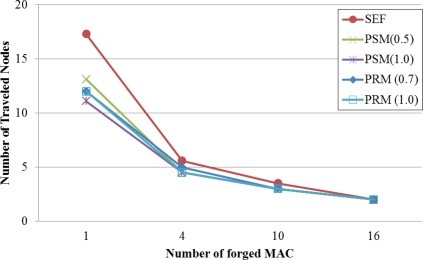
Average number of traveled nodes for filtering false reports with the security value (α).

Figure 4 shows an average number of traveled nodes to filter the false report. The reports are generated with 1, 4, 10, 16 forged MACs. The number of traveled nodes in the original SEF approach is the highest since routing paths are chosen with consideration of only hop counts. Though PSM detects the false reports earlier than PRM, the performance gap is acceptable.

**Figure 5.**
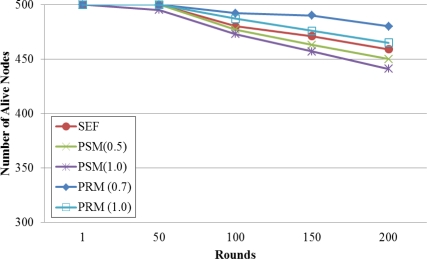
Number of alive node by elapsed time with the security value (α) in case that the number of forged MAC is 8.

Figure 5 shows the number of alive node that can send a data to next node or base station in SEF, PSM and PRM. We generate false reports that include eight forged MACs and inject the report into the network.

When the routing paths are established, each node considers hop counts or hop counts and partition information of keys. Therefore, the path would make the most secure paths in heavy traffic so that the nodes along the paths would consume more energy resources. Also, the super-node that has high in-degree (*i.e.*, many sub-nodes) would consume more energy resources. That is, the communication traffic of the super-node is more than others that have a low in-degree and the node should be consumes much energy than others. In other hand, PRM considers communication traffic when a sub-node selects a super-node. For these reasons, the network life time of PRM is better than that of either SEF and PSM.

## 7. Conclusion and Future Work

There are many security issues including false data injection attacks in WSNs. SEF [9] is the first solution that can alleviate the impacts of the attacks. While a sensing report is being forwarded toward the base station, the report is verified by the forwarding nodes. PSM [12] can enhance the detection power of SEF. Every control message stores the information on the filtering keys of the nodes it traveled on, when paths are established by flooding. A sensor node has an evaluation function to choose the most secure path based on the information.

In this paper, we proposed a path renewal method to provide WSNs with load balancing. A network is represented as a digraph and a communication traffic model for the network is proposed. Base on the model, an evaluation function to choose a new super-node is defined. The effectiveness of the propose method is shown with the simulation results. As future works, some AI algorithms will be applied in order to find further optimal solutions.

## Figures and Tables

**Table 1.** Migration of super-node.

SuperNode N_sup_; SubNode N_sub.1_, N_sub.2_, N_sub.3_;
IF N_sup.energy_ < TE THEN N_sup_ sends EM to N_sub.3_;
N_sub.3_ finds neighbor nodes;
IF neighbor nodes is NOT NULL AND fitness(neighbor nodes) > fitness(N_sup_) THEN Send FM to N_sup_; N_usb.3_ migrates to new SuperNode;
